# Delayed Diagnosis of Lumbosacral Lipomyelomeningocele With Tethered Cord: A Case Report

**DOI:** 10.7759/cureus.92310

**Published:** 2025-09-14

**Authors:** Fiorella G Rodriguez Campian, Alejandra C Vargas Castañeda, Mauricio D Puch Ramirez, Brigida Del Pilar G Tueros Salazar, Leslie I Torres Abono, Jesus S Luna, Carlos A Romero

**Affiliations:** 1 Faculty of Medicine, Universidad de Aquino Bolivia, Santa Cruz, BOL; 2 General Medicine, San Juan Bautista Private University, Lima, PER; 3 Neurological Surgery, Hospital de Niños Dr. Mario Ortíz Suárez, Santa Cruz, BOL; 4 General Medicine, Universidad Nacional Federico Villareal, Lima, PER; 5 General Medicine, Universidad de Aquino Bolivia, Santa Cruz, BOL; 6 Facultad de Medicina Humana, Universidad Nacional José Faustino Sánchez Carrión, Huacho, PER; 7 General Medicine, Prisma Health Greenville, South Carolina, USA

**Keywords:** closed neural tube defect, delayed diagnosis, equinovarus deformity, lipomyelomeningocele, lumbosacral mass, pediatric neurosurgery, spinal dysraphism, tethered cord syndrome

## Abstract

Lipomyelomeningocele (LMMC) is a rare form of closed spinal dysraphism arising from primary neurulation defects, characterized by a lipomatous mass that anchors the spinal cord. Early diagnosis is critical to prevent progressive neurological deterioration; however, in resource-limited settings, detection is often delayed. We report the case of a nine-year-old girl with a congenital lumbosacral mass identified at birth but underestimated during the initial neonatal assessment. Over time, she developed progressive gait disturbances and right equinovarus deformity. Magnetic resonance imaging performed later revealed a lumbosacral LMMC with tethered cord and occult spina bifida. The patient underwent a 15-hour surgical procedure consisting of LMMC excision and cord untethering, with no intraoperative complications. Postoperatively, she presented with preserved motor strength, residual hypoesthesia in the lower limbs, and bladder management with neuromodulation. At two-week follow-up, she showed stable neurological function, improved gait with rehabilitation, and satisfactory wound healing. This case underscores the importance of recognizing lumbosacral cutaneous stigmata as markers of occult spinal dysraphism, highlights the challenges associated with delayed diagnosis, and emphasizes the need for a multidisciplinary approach in the management of complex surgical cases. Furthermore, it reflects the global epidemiological burden of neural tube defects estimated at 18.6 per 10,000 live births and the disparities in diagnosis and access to specialized care in rural areas, reinforcing the importance of improving neonatal screening, the availability of imaging studies, and healthcare provider training to reduce preventable disability.

## Introduction

Lipomyelomeningocele (LMMC) is a rare form of closed spinal dysraphism characterized by a lipomatous mass that extends through a posterior vertebral defect, often causing tethered cord syndrome and progressive neurological deterioration if untreated. The global incidence of LMMC is estimated at three to six per 100,000 live births, with a higher prevalence in females; however, epidemiological data remain limited, particularly in low- and middle-income countries where access to specialized imaging and neurosurgical care is restricted [1].

Maternal risk factors such as folate deficiency, diabetes, and obesity are consistently associated with increased risk of neural tube defects, including LMMC. These findings emphasize the importance of adequate prenatal care and periconceptional metabolic control to reduce the incidence of such malformations [2]. The Apgar score, while primarily a measure of neonatal adaptation, has demonstrated correlations with long-term neurodevelopmental outcomes. Lower 5-minute Apgar scores, even moderately depressed, are associated with increased risk of neurological morbidity, highlighting the need for early monitoring in neonates with congenital spinal anomalies [3-5]. In resource-limited settings, delayed diagnosis of LMMC is common due to insufficient prenatal imaging, limited access to MRI, and scarcity of pediatric neurosurgical specialists. Such late recognition can complicate surgical anatomy, increase operative time, and elevate the risk of residual neurological deficits and orthopedic sequelae [1].

In this report, we present the case of a nine-year-old girl with a congenital lumbosacral mass and tethered cord diagnosed late, underscoring the interplay between maternal risk factors, delayed diagnosis, and surgical complexity in a resource-constrained environment. This case highlights the need for increased vigilance, improved prenatal care, and multidisciplinary management strategies to reduce preventable morbidity from LMMC [1,6].

## Case presentation

**Figure 1:**
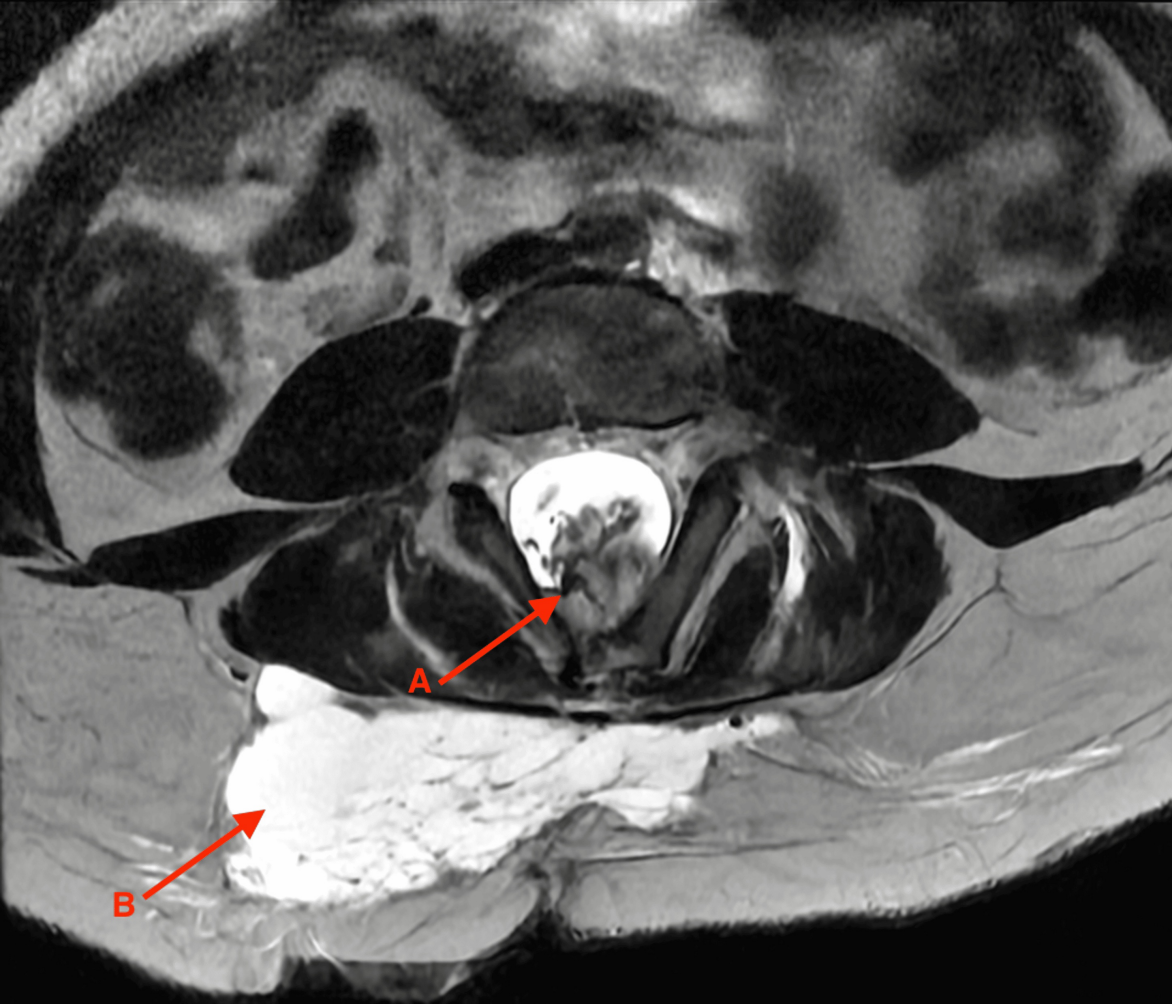
Axial lumbar magnetic resonance imaging A: Conus medullaris in continuity with hyperintense lipomatous tissue within the spinal canal. B: Hyperintense lipomatous tissue through a bony defect into the subcutaneous tissue, consistent with lipomyelomeningocele.

A nine-year-old female patient presented with a history of a congenital lumbosacral mass and progressive difficulty with standing and walking in May 2025. She was born at term by vaginal delivery, weighing 3 kg, measuring 50 cm in length, with a head circumference of 40 cm. Her Apgar score was 8 at one minute and 5 at five minutes, a situation that should have prompted close monitoring and neurological evaluation, which was not performed at that time. Prenatal studies were scarce and limited, and no central nervous system malformations were detected. Since birth, the mother had noticed a soft mass in the lumbosacral region, which was disregarded during the neonatal period. At the age of two, a general practitioner recommended a biopsy under the suspicion of a cutaneous lipoma; however, no histopathological report was obtained, and the approach was inadequate given the possibility of spinal dysraphism, reflecting the diagnostic limitations of the setting where she was evaluated. Over time, the mass showed progressive growth. At the age of six, the patient developed gait disturbances, internal rotation, and inversion of the right foot, as well as restrictions in active and passive mobility in the same limb. She was evaluated by orthopedics, where right clubfoot was diagnosed, and a lumbar magnetic resonance imaging (MRI) was requested. The study revealed a complex lipomyelomeningocele with tethered cord and occult spinal dysraphism. On axial T2-weighted sequences, a low-lying conus medullaris was observed in continuity with a hyperintense lipomatous mass herniating through a bony defect into the subcutaneous tissue (Figure 1).

**Figure 2:**
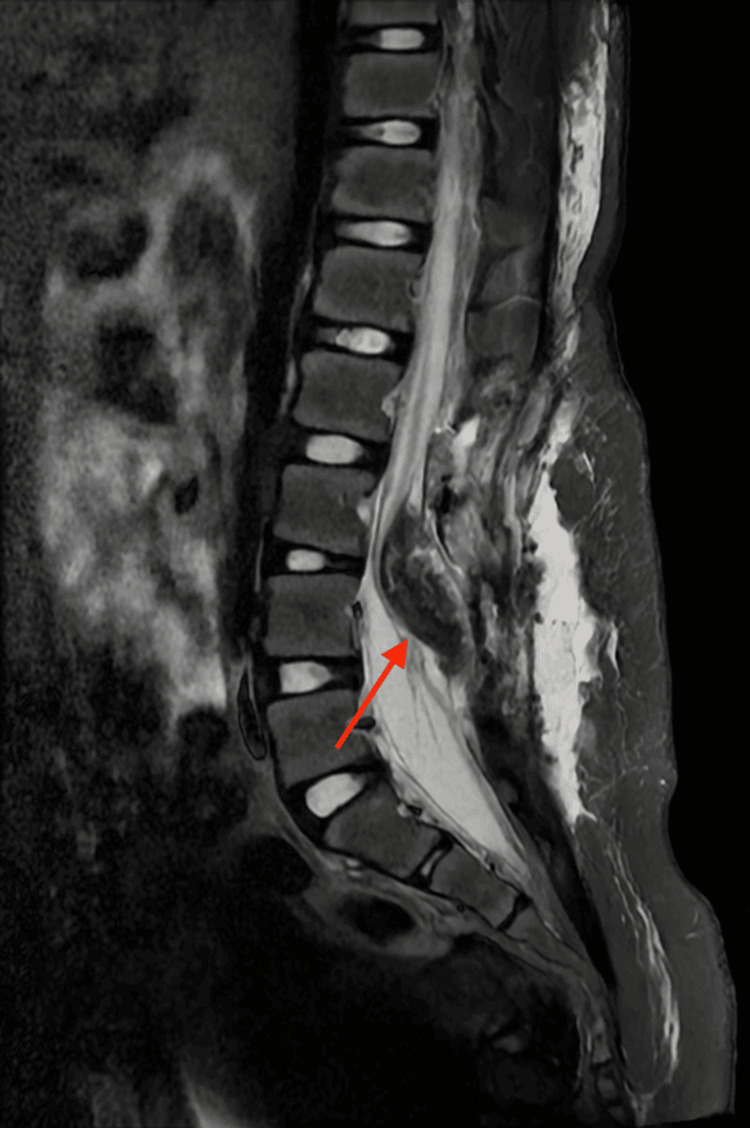
Sagittal lumbar magnetic resonance imaging Low-lying conus medullaris fused with hyperintense lipomatous tissue extending through a bony defect into the subcutaneous tissue, findings consistent with lipomyelomeningocele and tethered cord.

Sagittal images confirmed the low insertion of the conus medullaris (below L1-L2), fused with the lipoma (Figure 2).

**Figure 3:**
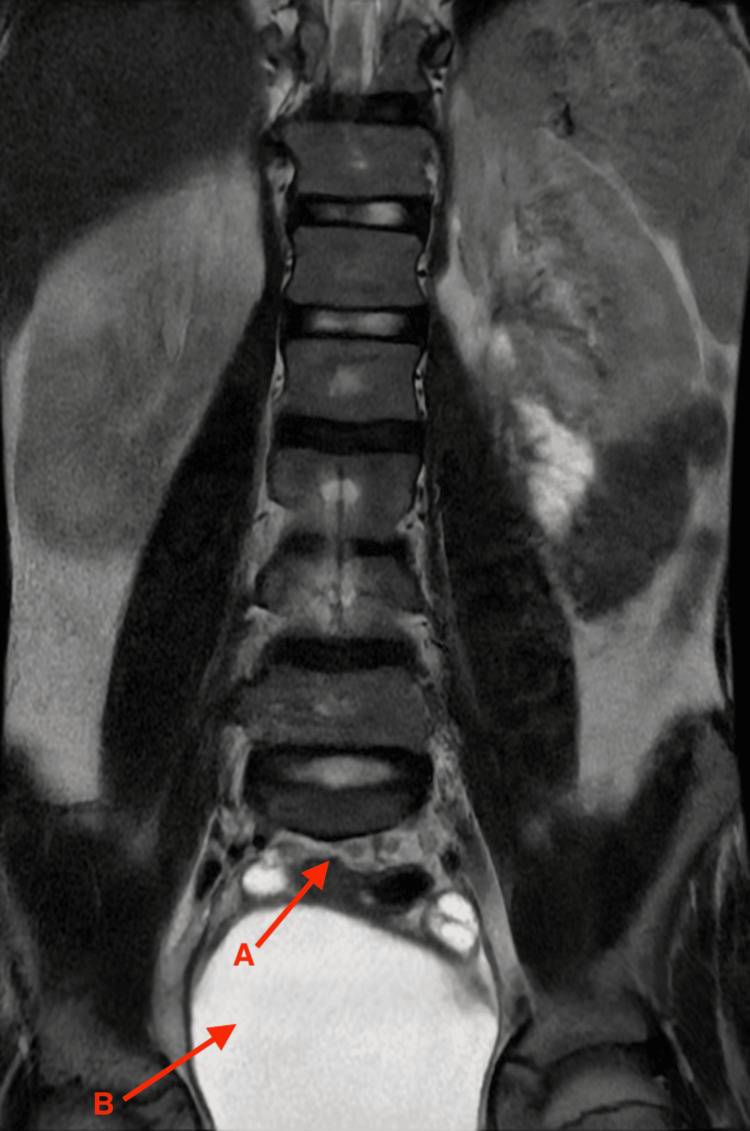
Coronal lumbar magnetic resonance imaging A: Midline bony defect with protrusion of hyperintense lipomatous tissue in continuity with the spinal canal. B: Extension of lipomatous tissue into the subcutaneous tissue, characteristic of lipomyelomeningocele.

Differentiation from normal subcutaneous fat was established based on the continuity of intradural lipomatous tissue with the spinal cord through the bony defect, a characteristic finding of lipomyelomeningocele (Figure 3).

On admission, the patient was in good general condition, conscious, afebrile, and without signs of intracranial hypertension. Neurological examination revealed decreased mobility of the right lower limb, associated with internal rotation and inversion of the foot, as well as plantar dysesthesias, more intense on the same side. No sphincter dysfunction was noted. On spinal examination, a soft, irregular, non-tender lumbosacral mass was identified, covered by a flat scar corresponding to the prior procedure. The presence of right clubfoot was confirmed. Patient underwent surgical resection of the lipomyelomeningocele, release of the tethered cord, L1-L2 laminectomy, and section of the filum terminale, with intraoperative neurophysiological monitoring. The surgery lasted 15 hours and 10 minutes, far exceeding the usual operative time due to the anatomical complexity of the lipoma and the need to preserve the nerve roots and spinal cord. No intraoperative complications were reported.

In the immediate postoperative period, she was somnolent, spontaneously ventilating with supplemental oxygen, with a Glasgow score of 13/15, afebrile, and hemodynamically stable. Bilateral plantar dysesthesias persisted (predominantly on the right), with preservation of motor strength in both lower limbs. The surgical wound evolved without complications, cerebrospinal fluid leakage, or infection. Management included clean intermittent catheterization, bladder neuromodulation therapy (understood as programmed functional electrical stimulation to optimize bladder emptying and prevent urological complications), meningitic-dose antibiotic prophylaxis, and strict lateral decubitus positioning. At two-week follow-up, the patient showed stable neurological function, improved gait with rehabilitation, and satisfactory wound healing. At two months, she maintained preserved motor strength, partial recovery of bladder function with ongoing neuromodulation therapy, and was ambulatory with orthotic support. No wound or infectious complications were noted during follow-up.

## Discussion

Lipomyelomeningocele (LMMC) is a rare form of closed spinal dysraphism that arises from abnormal primary neurulation and reflects a failure in neural tube closure during early embryogenesis. More broadly, neural tube defects (NTDs) remain an important congenital anomaly worldwide, with multifactorial origins that include both genetic and environmental influences. Among these, inadequate maternal folate status before and during early pregnancy has been consistently identified as the most significant and preventable risk factor, underscoring the importance of folic acid supplementation and food fortification programs in reducing their occurrence [6].

This condition results from abnormal primary neurulation with fatty/mesenchymal tissue continuous with the neural axis and protrusion through a posterior defect; MRI is the diagnostic modality of choice, and imaging protocols and reporting should follow contemporary radiologic consensus to ensure correct characterization before any invasive procedure is performed [7]. In practice, the continuity of intradural lipomatous tissue with the conus and its extension through a bony defect into subcutaneous tissue are the imaging hallmarks that differentiate LMMC from simple subcutaneous lipomas [7].

Timing of diagnosis is a major determinant of outcome. Large series and single-center reports show better functional results (ambulatory and urologic outcomes) when patients are identified and treated before progressive neurological deterioration or severe orthopedic deformity develops; delayed diagnosis increases surgical complexity, operative time, and the risk of poorer long-term function [8,9]. The present case illustrates how missed neonatal recognition and subsequent inappropriate local management (biopsy without prior MRI) contributed to delayed referral and an anatomically complex lesion that required prolonged operative time. International consensus emphasizes that cutaneous lumbosacral stigmata (midline mass, hairy patch, sinus, scar) mandate early imaging (ultrasound in neonates less than three months, MRI thereafter) prior to any biopsy or excision [7].

Anatomical and pathophysiological considerations are central to surgical strategy. Recent histopathological and anatomical work on the filum and conus demonstrates that the filum terminale and lipomatous tethering structures may contain neural elements, stretch-sensitive and nociceptive fibers, and embryonic remnants, explaining how tethering produces progressive neurological and urological dysfunction and why meticulous intradural dissection with neurophysiologic monitoring is required to preserve function [10,11]. These data support careful intraoperative mapping of rootlets and conservative preservation of functional neural tissue rather than aggressive “en bloc” resection when nerve roots are involved [8,9,12].

Surgical outcomes and complications. Contemporary pediatric series report that untethering and lipoma resection improve or stabilize neurological function in many patients, but outcomes depend heavily on preoperative neurologic status, patient age, and lipoma complexity; re-tethering (secondary adhesions/scar) remains an important long-term complication and is one reason for continuing surveillance after surgery [9,10,13]. Large retrospective cohorts underline that patients operated on after clinical deterioration have higher rates of residual deficits and orthopedic sequelae (e.g., persistent clubfoot), which is consistent with the prolonged operative time and complexity observed in this case [8,9].

The patient’s 5-minute Apgar score of 5 represents a moderately depressed value. Population studies in term infants show that lower 5-minute Apgar scores (particularly <4) are associated with increased risk of neonatal mortality and severe neurological morbidity; even moderately low 5-minute scores [13] correlate with higher odds of short- and long-term adverse outcomes compared with scores 9-10 [13,14]. Importantly, Apgar is a momentary physiologic score influenced by multiple perinatal factors (gestational age, intrapartum events, observer variability) and is not diagnostic for congenital structural lesions. In other words, a low 5-minute Apgar may reflect perinatal compromise that can worsen neurodevelopmental vulnerability, but it does not explain the embryologic origin of an LMMC; rather, in this case, the low Apgar is a relevant cofactor that should have prompted closer neonatal neurologic follow-up and earlier imaging, an opportunity that appears to have been missed [13,14].

Narrative and empirical analyses from Peru and other LMIC settings document geographic, economic, and system barriers to timely neurologic/neurosurgical care: limited access to diagnostic imaging (MRI), centralization of subspecialty services in urban centers, workforce shortages, and fragmented care pathways all contribute to late diagnosis and suboptimal early management for congenital neurologic conditions. The clinical history presents a paucity of prenatal studies, an early mass that was “disregarded,” and a biopsy recommendation without prior MRI is a pattern well described in the literature on health disparities and delayed access to specialist care [15].

## Conclusions

This case highlights three key lessons: first, lumbosacral cutaneous stigmata and low Apgar scores should prompt early imaging to rule out occult spinal dysraphism; second, invasive procedures such as biopsy must be avoided until appropriate imaging is obtained; and third, delayed diagnosis increases surgical complexity and the risk of permanent neurological and orthopedic sequelae, underscoring the importance of early referral to specialized centers.
